# Recent Advances in Metal-Based Antimicrobial Coatings for High-Touch Surfaces

**DOI:** 10.3390/ijms23031162

**Published:** 2022-01-21

**Authors:** Martin Birkett, Lynn Dover, Cecil Cherian Lukose, Abdul Wasy Zia, Murtaza M. Tambuwala, Ángel Serrano-Aroca

**Affiliations:** 1 Department of Mechanical and Construction Engineering, Northumbria University, Newcastle upon Tyne NE1 8ST, UK; c.c.lukose@nortthumbria.ac.uk (C.C.L.); abdul.zia@northumbria.ac.uk (A.W.Z.); 2 Department of Applied Sciences, Northumbria University, Newcastle upon Tyne NE1 8ST, UK; lynn.dover@northumbria.ac.uk; 3 School of Pharmacy and Pharmaceutical Science, Ulster University, Coleraine BT52 1SA, UK; m.tambuwala@ulster.ac.uk; 4 Biomaterials and Bioengineering Lab, Centro de Investigación Traslacional San Alberto Magno, Universidad Católica de Valencia San Vicente Mártir, c/Guillem de Castro 94, 46001 Valencia, Spain; angel.serrano@ucv.es

**Keywords:** antimicrobial, coating, high-touch surface, superhydrophobic, nanoprotrusion, SARS-CoV-2

## Abstract

International interest in metal-based antimicrobial coatings to control the spread of bacteria, fungi, and viruses via high contact human touch surfaces are growing at an exponential rate. This interest recently reached an all-time high with the outbreak of the deadly COVID-19 disease, which has already claimed the lives of more than 5 million people worldwide. This global pandemic has highlighted the major role that antimicrobial coatings can play in controlling the spread of deadly viruses such as SARS-CoV-2 and scientists and engineers are now working harder than ever to develop the next generation of antimicrobial materials. This article begins with a review of three discrete microorganism-killing phenomena of contact-killing surfaces, nanoprotrusions, and superhydrophobic surfaces. The antimicrobial properties of metals such as copper (Cu), silver (Ag), and zinc (Zn) are reviewed along with the effects of combining them with titanium dioxide (TiO_2_) to create a binary or ternary contact-killing surface coatings. The self-cleaning and bacterial resistance of purely structural superhydrophobic surfaces and the potential of physical surface nanoprotrusions to damage microbial cells are then considered. The article then gives a detailed discussion on recent advances in attempting to combine these individual phenomena to create super-antimicrobial metal-based coatings with binary or ternary killing potential against a broad range of microorganisms, including SARS-CoV-2, for high-touch surface applications such as hand rails, door plates, and water fittings on public transport and in healthcare, care home and leisure settings as well as personal protective equipment commonly used in hospitals and in the current COVID-19 pandemic.

## 1. Introduction

Although the challenge of microbial infection has been well met over the last century, the need to maintain adequate hygiene and control the transmission of infectious agents remains. Arguably, this threat is intensifying as novel viruses may enter the human population from animal reservoirs, as illustrated by the current global COVID-19 pandemic, and resistance to antimicrobial therapies develops especially with respect to microorganisms such as bacteria where its genetic determinants are rapidly exchanged between pathogens. Healthcare facilities represent important environments for infection transmission. Their users are often immunocompromised and are more susceptible to infection than the general population. Some of the bacteria encountered here are more likely to have developed or acquired antibiotic resistance. Within these healthcare settings, human high-touch surfaces such as clinical and housekeeping equipment, and fittings such as water taps, door plates, and bed rails, all have a high potential to spread deadly pathogenic infections. Similarly, in the more general environment, high-touch surfaces on public transport, non-domestic residential settings (care homes and hotels) and leisure facilities as well as personal protective equipment (PPE) such as face masks used for healthcare workers and many people in the current COVID-19 pandemic, present many possibilities for microbial transmission [1,2,3,4]. While hand washing, cleaning, and disinfecting can effectively mitigate the transmission of microbes, these solutions rely on human behaviour. In contrast, intrinsic antimicrobial surfaces offer a passive system that requires no human intervention, and their action is continuous rather than episodic. 

Therefore, antimicrobial high-touch surfaces have the potential to significantly reduce the lifetime of deadly microorganisms such as SARS-CoV-2, the coronavirus responsible for the COVID-19 pandemic. Contact killing surfaces containing Ag or Cu ions are proven to kill bacteria and inactivate viruses such as methicillin-resistant *Staphylococcus aureus* (MRSA), *Escherichia coli*, Influenza A, and Norovirus [5]. For instance, new research has found that a defined dose of SARS-CoV-2 could only survive for 4 h on Cu surfaces, whereas it was still detected on cardboard after 24 h and plastic and stainless-steel surfaces after three days [6]. Recently, the development of bioinspired nanoprotrusions has been suggested as an alternative method of contact-killing microorganisms. Ti nanopillars based on nanoprotrusions found on dragonfly wings have been shown to induce deformation and penetration of bacterial cell envelope and increased oxidative stress, which impedes bacterial growth [7]. Superhydrophobic surfaces are also renowned for their stable antibacterial properties. Structurally modified surfaces based on the lotus leaf [8], springtails [9] and termite wings [10] have become particularly desirable as stable antibacterial surfaces because their self-cleaning and water-resistant properties prohibit bacteria growth [11].

It is well documented that *contact-killing surfaces*, *nanoprotrusions,* and *superhydrophobic surfaces* all possess unique antimicrobial properties but recent research has highlighted the possibility of combining these phenomena to create materials with binary or ternary killing potential. This article begins with an overview of recent research into these individual phenomena before giving a detailed review of the latest advances and potential future synergies in combining these three discrete microorganism-killing solutions to create the next generation of super-antimicrobial metal-based coatings for high-touch surface applications.

## 2. Contact Killing Surfaces

**Figure 1:**
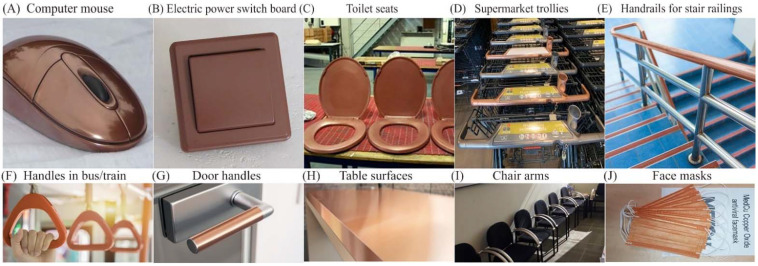
Cu-based antimicrobial coated commercial products. Adopted from public internet platforms: (**A**–**D**) [13], (**E**,**F**) [14], (**G**,**H**) [15], (**I**) [16], (**J**) [17].

The global antimicrobial coatings market size was valued at USD 8.34 billion in 2020 and is expected to grow at a CAGR of 13%, reaching USD 20.71 billion by 2028 [12]. Antimicrobial coatings of Cu, Ag, Zn, and TiO_2_ are proven to prevent frequently touched surfaces from serving as reservoirs for the spread of pathogenic microbes. They are used as antimicrobial coatings on products such as facemasks, supermarket trollies, door handles and push bars, passenger supports in buses and trains, touch screens and computer mice installed at public places, electric power switchboards, toilet seats, table and chairs contact surfaces, etc. Figure 1 presents some examples of commercial products made with a Cu coating to enhance their antimicrobial performance.

### 2.1. Copper

**Figure 2:**
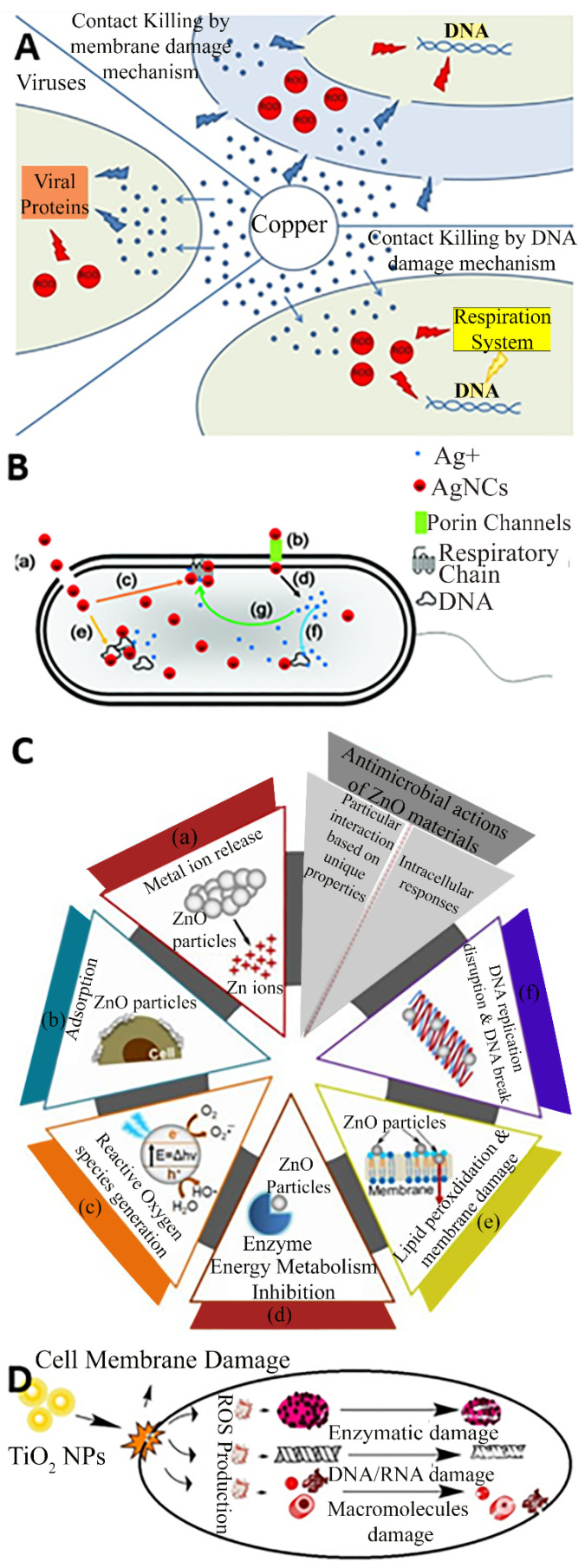
Contact killing mechanism of (**A**) Cu; (**B**) Ag; (**C**) ZnO and (**D**) TiO_2_. Reproduced with permission from: (**A**) [26], (**B**) [27], Adopted from (**C**) [28] (**D**) [29].

Cu is by far the most frequently used metal to create antimicrobial surfaces due to its efficiency in contact killing [18] and has been incorporated in its pure, alloy, composite, and nanoparticle forms in products such as door handles, handrails, and textiles [19,20]. Cu alloys are effective against a range of pathogens and have been proven to kill a very dense inoculum of 10^7^ *S. aureus* cells per ml within 10 min [21], and similar inocula of *Acinetobacter* spp. within 240 min [22] and *E. coli* O157 within 350 min [23]. Referring to Figure 2A, the antibacterial effect of Cu surfaces is attributed to a combination of the damage inferred by Cu(I) ions and reactive oxygen species (ROS), leading to lipid peroxidation and subsequent loss of membrane integrity, protein damage, DNA damage and cell death [24,25,26]. In general, other biocidal elements such as Ag, Zn, and TiO_2_ also express their toxicity via similar basic mechanisms where the bacteria are killed or deactivated by membrane rupture and damage leading to dysfunction of their fundamental components. For example, numerous mechanisms are associated with Ag nanoparticles and ions for antibacterial actions, such as membrane damage, and after diffusion into the cell, damage to respiratory chain components and DNA, causing dysfunction in replication as presented in Figure 2B [27]. Similarly, the common antibacterial mechanism for ZnO also involves ion release, adsorption, and the generation of reactive oxygen species, leading to membrane damage via lipid peroxidation, and ultimately the inhibition of metabolism and replication through damage to proteins and DNA as depicted in Figure 2C [28]. Figure 2D suggests that the TiO_2_ nanoparticles damage the cell membrane and reactive oxygen species are produced which damages the enzymes, DNA, RNA, and macromolecules to perform antimicrobial action [29]. 

Although Cu is a powerful antimicrobial agent, its killing efficiency depends on several important factors related to the form of the metal, like Cu concentration and nanoparticle size as well as environmental conditions like the temperature and relative humidity (RH) conditions under which the exposure occurs. Cu concentration is a key factor in alloy antimicrobial efficiency and surfaces containing a minimum of 55–70% Cu have been proven to effectively eliminate pathogenic microorganisms, like bacteria such as *S. aureus*, *E. coli*, *Enterococcus faecalis*, fungi such as *Candida albicans,* and viruses such as influenza viruses or the human immunodeficiency virus (HIV) [30]. Cu nanoparticle size is another important factor and several studies have shown that smaller size nanoparticles have better antimicrobial activity [31,32,33]. For example, Thekkae Padil and Cernik [31], found that small CuO nanoparticles of ~4.8 nm had significantly more potent antibacterial activity than larger nanoparticles of ~7.8 nm against both Gram-positive (*S. aureus)* and Gram-negative (*E. coli*) bacteria. Applerot et al. [33], demonstrated that these nanoparticles adhere to bacterial surface structures and due to the membrane damage created by the ROS generated, they may penetrate bacterial cells. However, reduction of CuO nanoparticles size can also lead to an increase in human cytotoxic effects and therefore needs careful consideration [34,35]. Environmental conditions can also have a significant influence on the antimicrobial effect of Cu surfaces. Ojeil et al. [36], showed that Cu alloys gave a >4-log reduction in *S. aureus* after 30 min at 37 °C and 100% RH. However, when conditions of 20 °C and 40% or 50% RH were imposed, the same effect took 60 min. This result highlights that Cu could offer adequate antimicrobial activity on hospital dry surfaces and even better performance in wet environments like water piping [18]. There have also been several studies reporting the antimicrobial effectiveness of Cu in a few minutes in dry conditions compared to several hours in wet conditions [37,38]. For example, Zhu et al. [37], found that Salmonella typhimurium resistant strains survived on Cu alloy surfaces for only 10 to 15 min in dry incubation instead of 0.5 to 2 h when moist incubation was performed, presumably, this is due to the imposition of the Cu insult in combination with what would be a stressful environment for the organism. Likewise, Cronobacter was killed within 1 min of drying on Cu alloys but took 10 min when kept moist.

These results confirm that high Cu concentration and small nanoparticle size, along with high temperature and variation in humidity are all important factors that could increase the antimicrobial activity of Cu-based coatings and surfaces.

**Figure 3:**
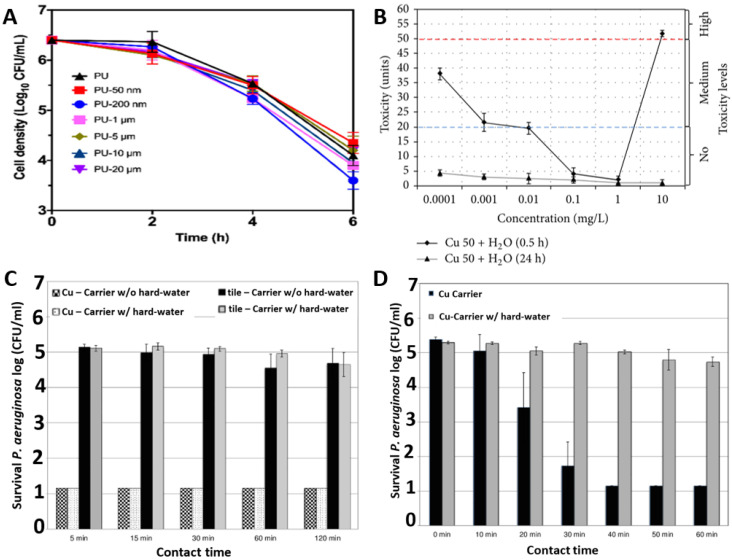
Role of Cu in antimicrobial activity: (**A**) effect of Cu particle size against *S. aureus* ATCC 25923; (**B**) effect of Cu concentration and humidity against *E. coli* M-17; (**C**) effect of dry and (**D**) moist environments against *P. aeruginosa* ATCC 15442. Reproduced with permissions: (**A**) [39], Adopted from: (**B**) [40], (**C**) and (**D**) [41].

Effective design of novel antimicrobial materials requires optimisation of the size and frequency of Cu nanoparticles inclusion. Figure 3A compares the kinetics of *S. aureus* death when exposed to C u-impregnated polyurethane materials as a function of Cu particles size. The Cu particle size was varied from 50 nm to 20 µm and the investigations demonstrate that 200 nm-sized Cu nanoparticles provided the most effective killing of the *S. aureus* bacteria [39]. Similarly, there appears to be a threshold concentration of Cu nanoparticles required to express potent toxicity to the bacteria. Figure 3B illustrates that the Cu nanoparticles at low concentrations between 0.01 mg/L to 1 mg/L are non-toxic for *E. coli* M-17. Otherwise a lower threshold range i.e., 0.0001 mg/L to 0.01 mg/L or excessive concentrations like above 1 mg/L gives a medium and high level of toxicity, respectively [40]. Figure 3C,D illustrate differential killing rates for *Pseudomonas aeruginosa* in dry and moist environments respectively. It can be seen that the antimicrobial performance of Cu has increased in a moist environment with a ~4-log reduction in 30 min for a moist environment, while it only reduced by ~0.5-log after 60 min in a dry environment [41]. 

### 2.2. Silver

Ag is also gaining popularity as an effective antimicrobial coating material. Its principal use is in the prevention of bacterial infections in open wounds and coating of various medical implants [42] and it has also been shown to reduce the levels of contaminant bacteria by up to 95.8% when used in healthcare environments to coat various products such as furniture, bedding, water taps, and signs, etc., [43,44]. 

Ag can be particularly effective when applied in coatings made of nanoparticles [45] and in nanostructures [46] containing large surface-to-bulk ratio particles with considerable quantities of Ag oxide that provide a source of Ag ions to effectively kill bacteria [47]. Ag nanoparticles are used as antimicrobial agents in a broad range of industrial applications including wound dressings, food and textile area, paints, household products, catheters, implants, and cosmetics, among many others [48]. Research has shown that the size of Ag nanoparticles can significantly influence their efficacy as an antiviral agent and the effective upper size limit appears to be around 25 nm [49]. Rogers et al. [50], showed that when Ag nanoparticles become too large they are unable to inhibit viral bonding to cell surfaces and may instead agglomerate and facilitate interaction between the virus and host cells. 

**Figure 4:**
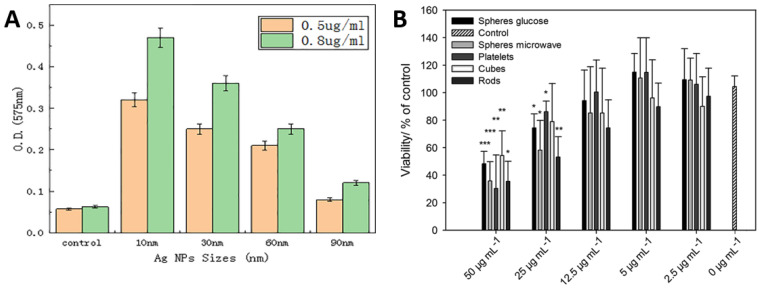
Role of Ag in antimicrobial activity: (**A**) effect of Ag nanoparticle size, suggesting 10 nm size gives optimum antimicrobial efficacy against *V. natriegens*; (**B**) effect of Ag concentration and morphology suggest that the concentration of Ag particles have a significant effect on the viability of mesenchymal stem cells. Whereas the morphology of Ag particles such as rod, sphere, cubes, etc., also influence cell viability but not as significantly as Ag concentration. Whereas, ( * ) presents the significance in differences when compared with control i.e., * *p* < 0.05; ** *p* < 0.005; and *** *p* < 0.001. Adapted from: (**A**) [51], (**B**) [52].

Referring to Figure 4A,B, it is deduced that the Ag nanoparticles should have optimized size and concentrations to deliver efficient antimicrobial actions. Figure 4A presents the optical densities used to estimate the amount of ROS generated in *Vibrio natriegens* after treating with different sizes of Ag particles. In a limited series of samples with Ag concentrations (0.5 and 0.8 μg/mL), there is an apparently consistent correlation between ROS generation and nanoparticle size; the highest yields of ROS arising from the smallest particles (10 nm) in both series with ROS yields being proportional to concentration [51]. Figure 4B presents that the cell viability decreases with increasing Ag particles concentration [52]. It is also worth noting that the morphology of the nanoparticle also influences the antimicrobial performance up to a certain level. 

Since Ag nanoparticles are less susceptible to surface oxidation than Cu in ambient conditions, they often exhibit greater antibacterial potency in vitro applications. Nevertheless, several previous studies have specifically investigated the potential of Ag-containing coatings to reduce microbial contamination of touch surfaces in healthcare settings [53,54,55]. For example, Taylor et al. [44], reported that the use of a Ag antimicrobial BioCote^®^ (Coventry, UK) coating technology gave a 95.8% reduction in bacterial population when compared to untreated surfaces. Hard, wear-resistant, Ag-ceramic coatings such as Ag-TiN [56,57,58], Ag-TaN [59], Ag-ZrN [60,61], and Ag-DLC [62] have also been widely studied for their antibacterial effects. Zhao et al. [57], studied the bactericidal properties of Ag-TiN coatings deposited by ion beam assisted deposition at modulation periods of 4 to 12 nm. They found that multi-layered films with a modulation period of 7.5 nm had no cytotoxic effect on mouse L292 cells and possessed the strongest bactericidal effect against *E. coli*. Hsieh et al. [59], prepared Ag-TaN coatings using a hybrid reactive co-sputtering and rapid thermal annealing method. Their results showed that both Ag particle size and total exposed Ag amount on the coating surface are critical parameters to ensure short-term antibacterial effects (<3 h). However, for longer-term (>24 h), the antibacterial efficiency is related to the Ag content within the Ag-TiN thin film. Kelly et al. [63], made a comparison of the tribological and antimicrobial properties of Ag-TiN, Ag-ZrN, and Ag-CrN nanocomposite coatings. They tested the coatings against *P. aeruginosa* and *S. aureus* and observed significant reductions in the number of viable cells with increasing Ag content, compared to the ‘pure’ nitride surfaces. Increasing Ag content also provided a reduction in the coefficient of friction but this was accompanied by reductions in hardness for all the coatings and wear resistance for some of the coatings. More recently (2021), Braceras et al. [64], also investigated Ag-TiN as an antimicrobial and wear-resistant coating in the range 4–66 at.% Ag. They showed some excellent results for antimicrobial activity but again this was counteracted by a gradual decrease in hardness with increasing Ag content in the films. In 2019, Mejia et al. [65], studied the combined influence of Ag/Cu nanoparticles on the microstructural and bactericidal properties of TiAlN-Ag/Cu coatings for medical applications deposited by magnetron sputtering. They evaluated the bactericidal effect of the coatings via in vitro inhibition and adhesion tests using *S. aureus* and *E. coli* and found that TiAlN-Ag/Cu with 17 and 20 at.% Ag/Cu exhibited a higher bactericidal effect than TiAlN, but this was accompanied by a 75% reduction in mechanical hardness.

### 2.3. Zinc

Zn is one of the essential biometals, as well as Fe, Cu, Mn, and Co and it is found in all human tissues [66]. It is another metal that has proven potent antimicrobial behaviour against a broad range of micro-organisms, including multidrug-resistant bacteria, and has shown great promise in biomedical applications [67,68]. Zn has demonstrated antiviral properties against a wide range of viral species and although the exact killing mechanisms are unclear, it appears to inhibit viral protease and RNA or DNA polymerase enzymatic processes, as well as physical processes such as viral attachment and uncoating [69]. The effectiveness of Zn as an antiviral agent was first demonstrated against the human rhinovirus in the form of Zn chloride, which provided a 99.99% reduction in the number of viral plaques (in vitro infections) formed [70]. Zn has also shown antiviral activity against all phases of the herpes simplex virus type 1 and type 2 lifecycles, including DNA polymerase function, protein production and processing, and inactivation of free viral particles [71,72,73]. 

When used for antimicrobial touch surfaces, Zn is usually combined with other metals to form an alloy or as ions within a coating structure. For example, Warnes et al. [74], observed that a change in the percentage of Zn, from 40 and 30% in a brass (CuZn) alloy, had a significant influence on the ability of the alloy to inactivate murine norovirus type 1, with alloys containing up to 30% Zn completely inactivating 5 × 10^5^ virus particles/cm^2^ within 2 h. Hodek et al. [75], investigated the antiviral properties of a hybrid coating containing Ag, Cu, and Zn cations against several viruses such as HIV type 1 (HIV-1), influenza, dengue virus, herpes simplex virus, and coxsackievirus. They measured a 99.5–100 % reduction in the infectious titer for HIV-1 after only 20 min of exposure to the coated slides, while slower virucidal kinetics were observed with other enveloped viruses, where 240 min of exposure to coated slides led to 97% (dengue), 100% (herpes simplex), and 77 % (influenza) reductions in virus titers. Interestingly, only marginal reductions in viral titer after 240 min of exposure were noticed for the non-enveloped coxsackie B3 virus.

**Figure 5:**
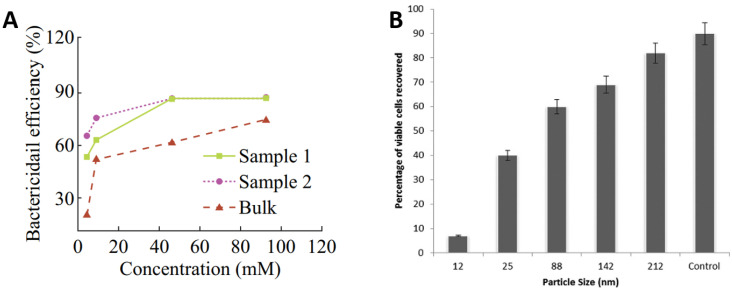
Role of ZnO in antimicrobial activity: (**A**) effect of ZnO concentrations of 0.1 mg/mL (sample 1), 0.3 mg/mL (sample 2), 0.5 mg/mL (Bulk) and (**B**) effect of ZnO nanoparticle size on antibacterial performance against *S. aureus*. Adapted from: (**A**) [76] Reproduced with permission from: (**B**) [80].

There has also been a vast number of studies on the use of Zn oxide (ZnO) nanoparticles and glasses as antibacterial agents. ZnO has been proven to be more toxic to bacteria in nanoparticle form rather than in micron equivalents [76,77] and is widely reported to demonstrate antimicrobial properties without the toxicity and environmental effects of other biocide agents like Ag, Cu, or their nanoparticles [78,79,80,81,82,83,84]. The strong bactericidal effect of ZnO nanoparticles is due to their ability to inhibit bacterial growth either by interacting with the bacterial surface or entering inside the bacterial cells [85]. Referring to Figure 5A, high concentrations of ZnO nanoparticles in water suspensions demonstrated elevated bactericidal efficiency for *E. coli* irrespective to their concentrations from 0.1 mg/mL (sample 1) to 0.3 mg/mL (sample 2) to 0.5 mg/mL (Bulk). However, significant improvements in efficiency were observed with increasing concentration up to 50 mM for sample 1 and sample 2, which become marginal hereafter. The bactericidal efficiency of bulk samples continues to increase more gradually with increasing concentration up to 100 mM [76]. Figure 5B presents the percentage of *S. aureus* viable cells recovered as a function of ZnO particle size. It can be seen that the control has the highest viable recovered cells, then large ZnO particles. The percentage recovery of viable cells reduces with reduced particle size. The studies suggest that the relative surface area of ZnO particles increased from 5.05 to 90.4 by reducing particle size from 212 to 12 nm, which is likely to increase the release of Zn ions for antimicrobial functioning [80].

It has been reported that ZnO nanoparticles show selective toxicity with regard to prokaryotic (bacterial) and eukaryotic (human and fungal) systems being more toxic for the prokaryotic cells [86]; i.e., both Gram-negative and Gram-positive bacteria are killed with lower ZnO concentrations than human T cells [87]. Esteban-Tejada et al. [88], investigated the antibacterial and antifungal activity of a new family of non-toxic ZnO-containing glasses. The transparent glasses with a ZnO content of 15 to 40 wt.% were shown to be biocompatible and chemically stable in different media and demonstrated excellent biocidal activity against both Gram-negative (*E*. *coli*) and Gram-positive (*S*. *aureus*) bacteria and yeast (*Candida krusei*). Ragupathi et al. [80], studied the size dependent bacterial growth inhibition and mechanism of antibacterial activity of ZnO nanoparticles. They found that antibacterial activity against *S. aureus* was inversely proportional to the size of the ZnO nanoparticles and that it might involve both the production of ROS and the accumulation of nanoparticles in the cytoplasm or on the outer surfaces of the cell surfaces. It is clear from these results that ZnO nanoparticles have the potential to be developed as antibacterial agents against a wide range of microorganisms.

### 2.4. Titanium Dioxide

TiO_2_ has attracted much attention for its photocatalytic properties and its resultant applications to the inactivation of bacteria, fungi, and viruses, owing to its low cost, biocompatibility, and corrosion resistance [89]. 

There have been several recent studies on TiO_2_ antimicrobial coatings. For example, Nakano et al. [90], tested the antibacterial activity of TiO_2_ coatings with a range of bacteria and found that all tested strains were photocatalytically inactivated under UVA exposure. More recently, Khaiboullina et al. [91], demonstrated the inactivation of human coronavirus HCoV-NL63 using TiO_2_ nanoparticle coatings in various humid environments under UV radiation, while Yoshizawa et al. [92], demonstrated the photocatalytic activity of TiO_2_ coatings against a bovine coronavirus under visible light irradiation of 500 lx. 

Creating binary or ternary coatings of TiO_2_ co-loaded with other metals like Cu and Ag has been shown to demonstrate even higher viral inactivation compared to coatings that contain only TiO_2_. For example, Negrete et al. [93], reported the efficacy of Ag-TiO_2_ coated surfaces against vesicular stomatitis virus (a safe surrogate of SARS-CoV-2) under visible light irradiation, while Moongraksathum et al. [94], showed that a 1 wt.% concentration of Ag in a TiO_2_ composite coating outperformed a simple TiO_2_ coating in terms of antibacterial effectiveness by more than 6 times when testing against influenza A and enterovirus. Furthermore, Rao et al. [95] showed that loading TiO_2_ nanowire membranes with Ag and Cu increased the disinfection of drinking water in comparison to TiO_2_ alone or in combination with just one metal. They demonstrated a 4-log reduction in bacteriophage MS2 activity for Cu–Ag–TiO_2_ in the UV light irradiation condition compared to less than 3-log reduction for TiO_2_ alone, a difference that was attributed directly to the co-loading of Ag and Cu. Environmentally benign TiO_2_ photocatalytic coatings with active ingredients are one of the best options to provide long-term antimicrobial efficiency with anti-corrosion surface features. Coatings of TiO_2_ containing active metals like Cu and Ag could demonstrate the maximum microbial disinfection efficacy compared to bare metals of Cu or Ag or TiO_2_ [89].

**Figure 6:**
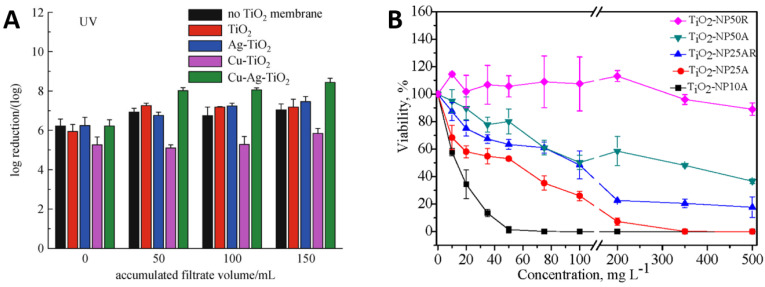
(**A**) performance of TiO_2_ nanoparticles in combination with Cu and Ag particles and (**B**) influence of TiO_2_ particle size and concentration on *E. coli* viability. Reproduced with permission from: (**A**) [95], Adapted from: (**B**) [96].

Figure 6A illustrates the inactivation of *E. coli* by TiO_2_ and its composites with Ag and Cu under ultraviolet light. The TiO_2_ particles showed high performance alone and the addition of Ag and Cu significantly increased this performance [95]. Figure 6B reports bacterial killing as a function of TiO_2_ particle size and concentrations. It can be seen that the bacterial viability reduces with increasing TiO_2_ particle concentration irrespective of the particle size, but small particles of 10 nm size kill more effectively than those of 50 nm size. Similarly, smaller TiO_2_ particles were more effective at lower concentrations such as 50 mg/L of 10 nm particles to kill 100% of bacteria, whereas the bacterial viability remained around 90% for larger TiO_2_ particles of 50 nm size, even at higher concentrations of 500 mg/L. It can be deduced that small TiO_2_ nanoparticles with lower concentrations may outperform large particles at higher concentrations [96].

### 2.5. Nanoparticle Synthesis

Table 1 presents the common synthesis methods and their sub-types to produce Cu, Ag, ZnO, and TiO_2_ nanoparticles. Generally, these antimicrobial nanoparticles are produced with chemical, physical, and biological methods. Chemical methods normally include sol-gel, reduction and reactions, microemulsion, sonochemical, electrochemical, chemical vapor deposition, and hydrothermal methods. Physical methods usually include laser ablation, evaporation, arc discharge, ball milling, etc. Whereas biological methods are a green synthesis route that is based on bacteria, viruses, fungi, algae, and plant-based extracts to synthesize nanoparticles. The chemical methods are simple, scalable, and give good control on growth mechanisms, however, the disadvantages are hazardous and flammable chemicals, expensive surfactants, and small yields. Physical methods are usually fast and environmentally friendly, but they need high energy inputs and expensive instrumentation. Biological methods provide good purity and localized synthesis but there is limited knowledge available to understand unknown mechanisms. In addition, DNA metallization [97,98], DNA programmed growth for produced biofunctionalized nanocrystals [99] are also reported for antimicrobial applications. Table 1 describes the advantages and disadvantages of each method associated with Cu, Ag, ZnO, and TiO_2_ nanoparticle synthesis.

The antimicrobial efficacy of biocidal nanoparticles depends on their size, morphologies, concentrations, pH, contact time, humidity, temperature, type, and amount of microorganisms [100]. Referring to dimensional features of biocidal nanoparticles, high-performance antimicrobial actions are desirous to have a high surface area to mass ratio to release more ions for contact killing actions [101]. Whereas it is well recognized that the morphology and therefore toxicity of nanoparticles depend on the synthesis methods [102,103]. Hence, there are no definite values for threshold size and concentration of Cu, Ag, ZnO, and TiO_2_ nanoparticles for superior antimicrobial action. A confined set of literature reviewed in this article suggests that Cu nanoparticles of 200 nm size have demonstrated superior performance against *S. aureus* [39] while 50 nm-sized Cu nanoparticles with 1 mgL^−1^ concentration have outperformed against *E. coli* [40] bacteria and a 30 min contact time of Cu nanoparticles has demonstrated 4-log reduction of *P. aeruginosa* [41]. Similarly, 10 nm-sized Ag nanoparticles have shown the best antimicrobial actions against *V. natriegens* [51] while a 5 µgL^−1^ [52] has been observed as the optimum concentration for the viability of mesenchymal stem cells, irrespective of particle morphology. The lower concentrations (10 µg/mL) of Ag nanoparticles in composition with graphene oxides have also shown superior antimicrobial performance [104]. In the same way, ZnO nanoparticles have shown an optimum concentration of 300 mgL^−1^ [76], and the antimicrobial performance increases with reducing particle size [80]. Similarly, 10 nm particle size with 50 mg/L^−1^ concentration [96] has been found as the optimum value for TiO_2_ nanoparticles among the literature studies investigated here.

## 3. Superhydrophobic Surfaces

**Figure 7:**
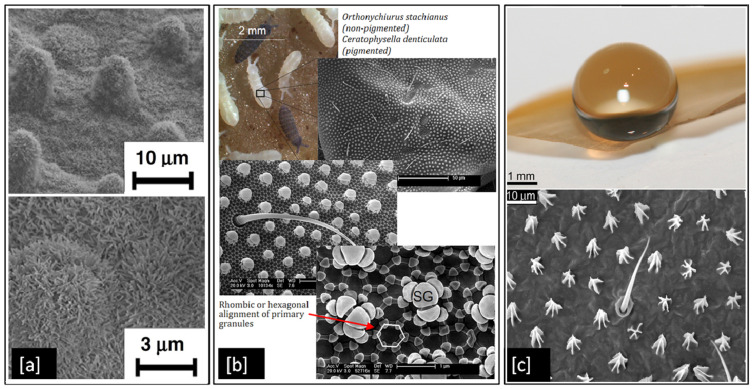
Examples of superhydrophobic surfaces in nature: (**a**) SEM image of lotus leaf at two different magnifications; (**b**) SEM images of springtail showing hierarchical structures and (**c**) Optical image of an interaction of a 10 μL water droplet on a termite wing and a topographical view of the wing membrane. Reproduced with permission from: (**a**) [8], Adapted from: (**b**) [9], (**c**) [10].

Although metals like Ag and Cu have proven antibacterial properties, they can become ineffective over time as bacteria develop resistance to them [150]. However, superhydrophobic surfaces could present a long-lasting solution to control the spread of infectious bacteria as they are purely structural and do not develop bacterial resistance. They are particularly desirable as antibacterial surfaces because of their ability to self-clean [11], by repelling water from their surface due to large contact angles > 150° between the liquid droplets and the surface itself [151]. There are several examples of superhydrophobic surfaces in nature such as the lotus leaf [8], springtails (*Collembola*, *Entognatha*) [9], and termite wings (*Nasutitermes* sp.) [10], which demonstrate properties such as self-cleaning, and flight efficiency. These superhydrophobic surfaces result from hierarchically aligned nanoscale structural elements, which minimise the contact area between the surface and liquids or particles [9]. The alignment nature of hierarchical structures varies from species to species. For example, in Figure 7a, we can see micron-sized mounds with nano-scaled hair-like structure distributed on the lotus leaf surface [8], in contrast to Figure 7b, which shows secondary granules uniformly spread across hexagonally arranged primary granules on a springtail surface [9], or in Figure 7c, where termite wing membranes can be seen with micrasters and microtrichia spread in a sheet like structure [10].

There have been numerous attempts to mimic such superhydrophobic behaviour artificially through structural alterations of nanomaterial and coating surfaces using techniques such as sol–gel and chemical processing [152,153], electrospinning [154], laser or plasma treatment [155,156,157], layer-by-layer deposition, photolithography, colloidal self-assembly, chemical vapour deposition (CVD), and physical vapour deposition (PVD) [158]. 

**Figure 8:**
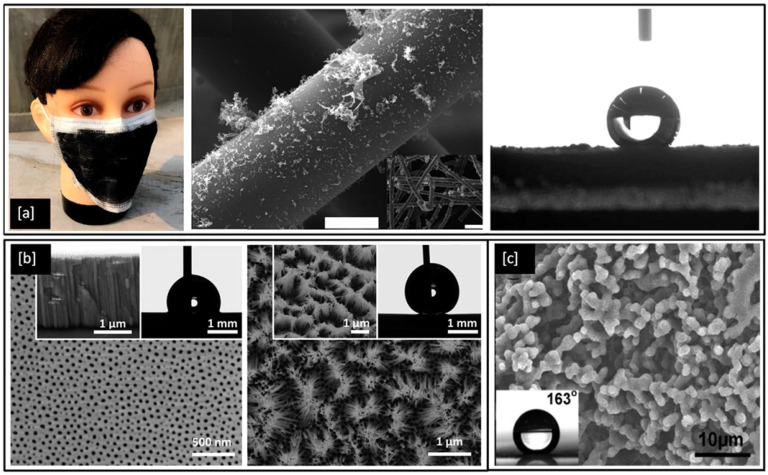
(**a**) Optical and SEM images of laser-fabricated graphene mask (scale bar = 10 μm) and the resulting water contact angle of 141° on the mask; (**b**) FE-SEM images of hydrophobic nanoporous Anodic Aluminium Oxide (AAO) transformed into nano pillared AAO with extended post etching (inset shows improvement in water contact angle) and (**c**) SEM image of a film cast from a block copolymer micelle solution (0.01 g/mL) (inset shows the water contact angle > 160°). Reproduced with permission from: (**a**) [161], (**b**) [162], (**c**) [164].

Superhydrophobic surfaces and coatings have been developed for a range of applications such as industrial and medical devices, self-cleaning of windshields and antennas, de-icing of glass surfaces, outdoor textiles, antibacterial and antifouling surfaces for biomedical and marine industries [159] as well as personal protective equipment kits, such as face shields and facemasks to fight the COVID-19 pandemic [160,161]. One such example is shown in Figure 8a, an optical image of a laser fabricated graphene mask, an artificially made superhydrophobic surface with graphene-coated on its non-woven fibre which helps the mask to exhibit a contact angle greater than 140°, thereby repelling the virus-carrying droplet away from nasal and oral cavities [161]. However, methods for the fabrication of such superhydrophobic and antimicrobial metal surfaces are still relatively limited. In 2017, Hizal et al. [162], engineered nanoporous and nanopillared hydrophobic aluminium surfaces by anodizing and post etching processes and showed a significant reduction in adhesion for *S. aureus* and *E. coli* bacteria. The surface features play a critical role in increasing the superhydrophicity of the surface. Teflon coated hydrophobic surfaces of anodic aluminium oxide (contact angle < 120°) depicts nanoporous surface features in its FE-SEM images as shown in Figure 8b but with prolonged etching, the surface is seen to be transformed into nanopillared structures which exhibit contact angles greater than 160° and become superhydrophobic in nature [162]. In 2018, Bartlet et al. [163], studied the antibacterial activity of superhydrophobic titania nanotube arrays prepared by anodizing and chemically etching Ti, which also reduced bacterial attachment on the surface. 

**Figure 9:**
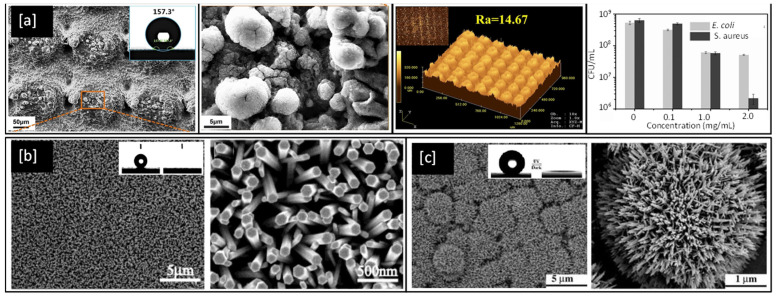
(**a**) SEM images of a bare Stainless Steel substrate, laser-etched and with the PDA@ODA modification (inset shows water contact angles), corresponding high magnification and Laser confocal microscopy images, and plots of colony-forming units per mL (CFU/mL) for *E. coli* and *S. aureus* bacteria with a concentration of PDA@ODA compound [Reproduced with permission from reference no. S.Li et al., 2019] (**b**) As-prepared ZnO nanorod films at low and high magnifications (inset shows the water droplet shape before (left) and after (right) UV illumination) [Reproduced with permission from reference no. X.Feng et al., 2003] (**c**) Low and High magnification images of TiO_2_ nanorod film (inset shows contact angle of 154°). Reproduced with permission from: (**a**) [165], (**b**) [166], (**c**) [167].

In 2019, Li et al. [165], investigated biomimetic superhydrophobic stainless surfaces prepared by a two-step method of laser interference patterning and in-situ polymerization, which successfully realized the state transition from superhydrophilic to superhydrophobic while simultaneously improving antimicrobial behaviour. SEM and laser confocal microscopy images of sample surfaces from Li et al.’s work are shown in Figure 9a and bear a close resemblance to the natural surface of the lotus leaf and exhibit water contact angles better than 155°. Similarly, FE-SEM images of nanorod structures of Zn and Ti oxides developed by Feng et al. can be seen in Figure 9b,c respectively and are observed to exhibit water contact angles greater than 150° and are therefore superhydrophobic in natural [166,167].

## 4. Nanoprotrusions

**Figure 10:**
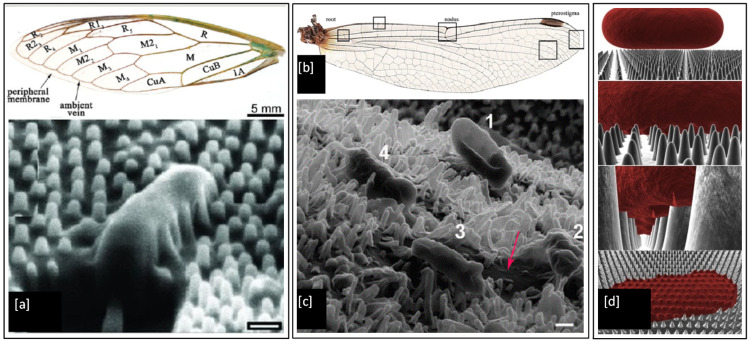
(**a**) Structural physiology of the forewing of cicada and SEM image of a *P. aeruginosa* cell sinking between the nanopillars on the wing surface (scale bar = 200 nm); (**b**) Optical image of wings from common sanddragon dragonfly; (**c**). SEM images of four *E. coli* bacteria attached to the uncoated nanopillar surface of a dragonfly wing in progressive stages of death and a red arrow marking the darker region formed by leakage of cellular fluid flooding the nanopillars (scale bar = 200 nm) and (**d**) 3-D biophysical model of the interactions between cicada wing nanopillars and rod-shaped bacterial cells. Reproduced with permission from: (**a**) [168], (**b**) [178], (**c**) [171], (**d**) [179].

It is well known that the wings of insects such as the cicada and dragonfly possess antibacterial properties. It has been shown that physical nanoprotrusions found on the wing surface stretch and damage the microbial cell upon contact, leading to lysis and death [168,169,170,171,172,173]. This effect was first noticed on the cicada wing, as shown in Figure 10a, where individual Gram-negative bacteria (*P. aeruginosa*, *Branhamella catarrhalis*, *E. coli*, and *Pseudomonas fluorescens*) cells have been observed to sink and spread between nanopillars of the wing surface, resulting in attachment and mechanical rupture of the bacterial cell wall, leading to death within 20 min [168]. The morphology of the cells did not appear to play any role in determining cell susceptibility. The bactericidal activity of the wing was also found to be quite efficient with ~6 × 10^6^ *P. aeruginosa* cells in suspension inactivated per square centimetre of wing surface after 30 min of incubation [173]. Dragonfly wings have also been shown to mediate the killing of Gram-negative (*P. aeruginosa*) and Gram-positive (*S. aureus* and *Bacillus subtilis*) bacteria. The capillary architecture of the nanoprotrusions present in the dragonfly wing leads to enhanced cell wall stress and deformation, causing cell wall rupture and leakage of cytosol fluid from cells, which can be seen as darker colour flooding in between the nanopillars marked by a red arrow in Figure 10c [171]. These unique bactericidal properties of cicada and dragonfly wings are of significant interest to researchers [174,175,176,177], as the physical nature of bacterial killing of these natural surfaces could provide an effective strategy to prevent biofilm formation and inspire the next generation of synthetic antimicrobial touch surfaces.

**Figure 11:**
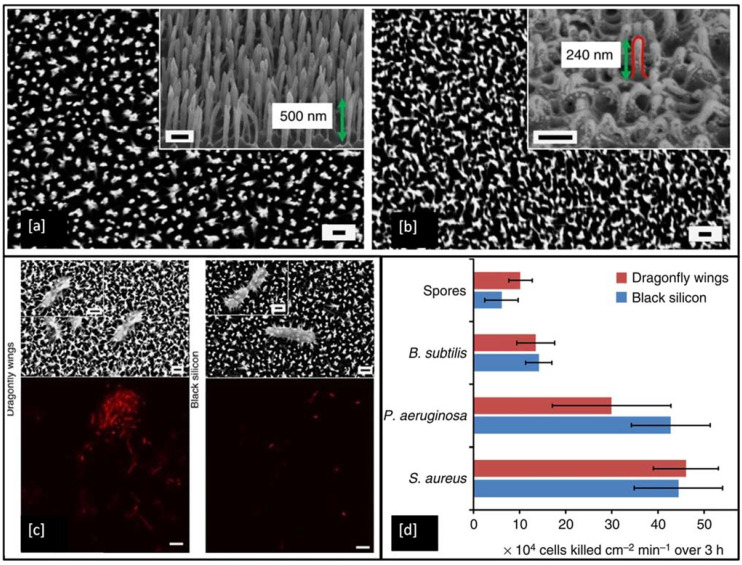
SEM images showing surface patterns of (**a**) black Si and (**b**) dragonfly forewings (measured nanopillars highlighted by red line, magnification = 35 k, scale bar = 200 nm), (**c**) SEM images and confocal laser scanning micrographs show *P. aeruginosa* cells are significantly disrupted through interaction with both the dragonfly wing and black Si (scale bars = 200 nm) and (**d**) Bactericidal efficiency of black Si and dragonfly wings on various bacterial strains. Reproduced with permission from [169].

To date, a wide range of nanofabrication techniques has been utilised to generate bactericidal nanotopographies on synthetic materials, including black silicon (bSi) [169], Ti [180], Ti alloy [181], and polymers [182]. Figure 11a shows an example of artificially made black silicone, bearing a strong resemblance to the surface features of the dragonfly wing (Figure 11b), but the black silicone nanopillars are made double in height and are sharper when compared to the features on the dragonfly wing. In the antimicrobial test, the *P. aeruginosa*, *S. aureus*, *B. subtilis*, appeared to be significantly disrupted when they interacted with both of these surfaces. Encouragingly, the very resilient endospores of *B. subtilis* were also inactivated by the material. The non-viable bacterial cells stained with propidium iodide (red) in Figure 11c shows that under confocal laser microscopy all of the cells appeared red, indicating the high efficiency of these surfaces in inactivating the bacteria [169].

There are a number of different models for the process of contact killing. The biophysical model proposes that bacteria cell membranes are physically stretched and ruptured upon contact with nanopillars on the cicada wing [179]. The elastic mechanical model suggests that Gram-positive bacteria have a lower maximum stretching capacity and are therefore less susceptible to nanopillar deformation and rupture but antibacterial properties can be enhanced by increasing the spacing and sharpness of the nanopillars [183]. The quantitative thermodynamic model proposes that the antibacterial efficacy of nanopatterned surfaces is governed by a balance between adhesion energy and deformation energy and the degree of cell envelope stretching can be enhanced by increasing nanopillar spacing to 100 nm and reducing nanopillar diameters to 50 nm [184].

**Figure 12:**
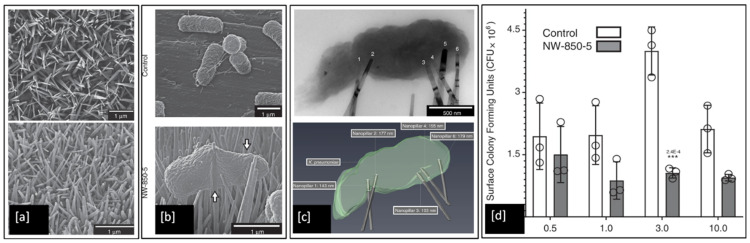
SEM images of (**a**) TiO_2_ nanopillar surface developed on Ti_6_Al_4_V substrate; (**b**) *K. pneumoniae* bacteria envelope interaction with Ti alloy control compared to deformation induced by TiO_2_ nanopillar surface; (**c**) Brightfield TEM and a 3D reconstruction of the tomogram, showing multiple nanopillars penetrating the bacterial envelope; and (**d**) Colony-forming units (CFU) determined for *K. pneumoniae* when incubated on the Ti alloy control or TiO_2_ nanopillar surfaces for up to 10 h (*** indicates *p* ≤ 0.001 relative to control, as determined by one-way ANOVA and Tukey-HSD post hoc test). Adapted from [7].

Although numerous models have proposed that the contact killing ability of both natural and synthetic nanopillars is due to mechanical rupture of the bacterial cell envelope, this has not been demonstrated conclusively and the precise bacteria-killing mechanism remains unclear. However, a very recent study by Jenkins et al. [7], investigated the antibacterial effects of dragonfly-inspired TiO_2_ nanopillar surfaces grown on Ti_6_Al_4_V alloy by thermal oxidation. SEM micrographs of the TiO_2_ nanopillars in Figure 12a can be clearly seen to induce deformation (white arrows in Figure 12b) in the outer membrane of the *Klebsiella pneumoniae* (Gram-negative) bacteria compared to the flatter Ti_6_Al_4_V alloy control sample. They also utilised electron tomography techniques to reconstruct detailed 3D visualisations of bacteria adhered to nanopillars, see Figure 12c, and discovered that the mechanistic basis of contact killing is multifactorial and nanotopography dependent. While deformation and subsequent penetration of the bacterial envelope by nanopillars were confirmed, these mechanisms did not result in mechanical rupture or cell lysis. Furthermore, using assays of bacterial viability they also identified a nanopillar-induced cell impedance, which is expected to reduce the capacity of bacteria to replicate on nanopillar surfaces, and thus could enhance the antibiofilm properties of nanopillar surfaces. Additionally, their analysis showed that oxidative stresses induced within bacterial cells upon contact with nanopillars, inhibit bacterial growth and biofilm formation, leading to time-dependent reductions in bacterial viability.

## 5. Recent Advances in Antimicrobial Coatings and Surfaces

It is clear from the above review that contact-killing surfaces, superhydrophobic surfaces, and nanoprotrusions all possess their own unique bacteria-killing potential. It is therefore not surprising that some of the most recent advances in metal-based antimicrobial coatings and surfaces focus on combining two or more of these microorganism-killing phenomena. 

**Figure 13:**
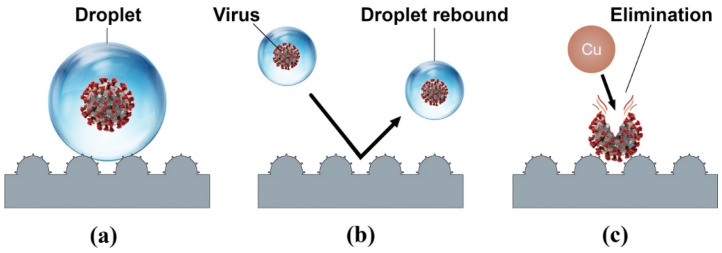
A schematic of our proposed transmission combating strategy: (**a**) Virus encapsulation, (**b**) contamination suppression, and (**c**) virus elimination. Reproduced with permission from [185].

The vast majority of recent studies have concentrated on the combined antimicrobial effects of superhydrophobic surfaces and chemical contact-killing surfaces of Cu, Ag, Zn, or TiO_2_. There have been several attempts to create superhydrophobic Cu-based antimicrobial surfaces [160,185,186,187,188]. Meguid and Elzaabalwy [185] proposed a flexible superhydrophobic silicone/epoxy-based nanocomposite coating containing hydrophobic silica nanoparticles and biocidal Cu nanoparticles, to combat COVID-19 using a 3-step strategy, see Figure 13. Firstly, exposure to virus encapsulation is reduced as droplets exist in a Cassie-Baxter wetting state [189] and maintain their spherical shape upon contact with the superhydrophobic surface (Figure 13a). Secondly, contamination is suppressed as droplets containing the virus either rebound or roll off the superhydrophobic surface (Figure 13b). Finally, any remaining traces of the virus on the surface are eliminated by the antimicrobial properties of the Cu nanoparticles within the nanocomposite surface (Figure 13c). Their results to date [160], have revealed that the developed silicone/epoxy-based nanocomposite coatings can maintain their superhydrophobicity under a range of harsh conditions such as elevated temperatures, chemical exposure to sulphuric acid or sodium hydroxide, and mechanical abrasion. However, the antimicrobial performance of these coatings is yet to be fully established and there could be concerns that microbes will be expelled from the surface rather than being effectively inactivated, especially if the technology is to be scaled up to create a large-scale antimicrobial surface.

**Figure 14:**
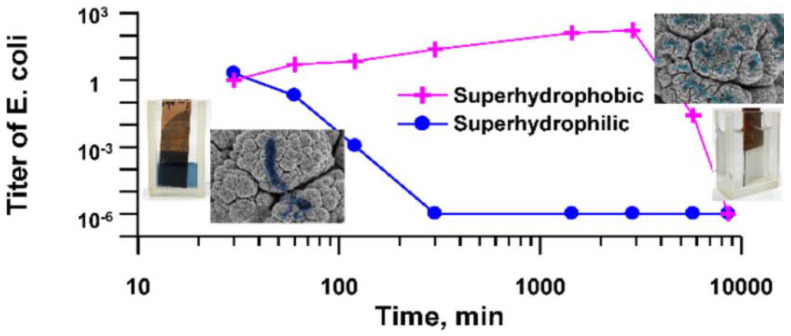
Comparison of the *E. coli* killing effect over time seen for superhydrophobic and superhydrophilic Cu surfaces prepared by nanosecond laser processing. Reproduced with permission from [186].

Boinovich et al. [186], compared the bactericidal activity of superhydrophilic and superhydrophobic Cu surfaces prepared by nanosecond laser processing against *E. coli* and *K. pneumoniae* bacteria. They found that the hierarchically roughened surfaces were able to effectively kill any cells in direct contact with it via a process of piercing, deformation, and cell membrane damage. There was also a significant difference in the bactericidal action of the Cu surfaces with variation in substrate wettability and the superhydrophilic surface gave the highest bactericidal effect due to the larger contact area between the bacterial dispersion and corrosive Cu ions. In contrast, the superhydrophobic surface initially showed improved corrosion resistance with weak cell adhesion and low levels of Cu ions in the bacterial cells in aqueous dispersion. However, a gradual transition from the superhydrophobic state to a hydrophilic one triggers the Cu ion bacteria cell killing mechanism, seen for the superhydrophilic surfaces, see Figure 14.

**Figure 15:**
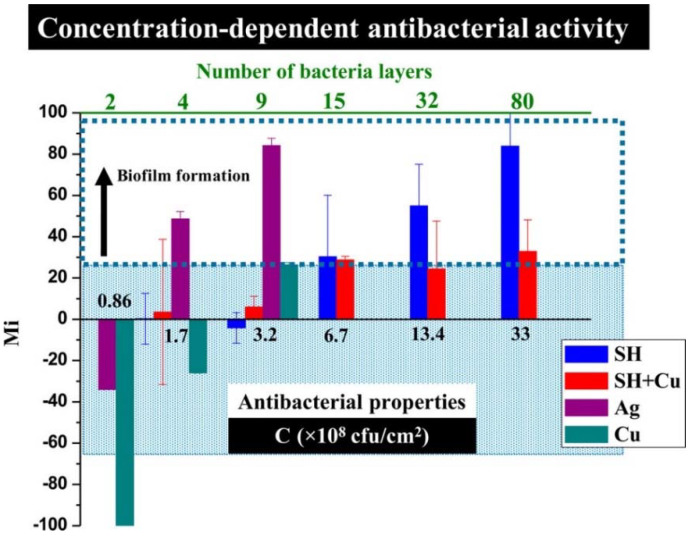
Effect of the bacteria surface density on the antibacterial activity for the 20th day of the test. *M*_i_ represents the change in fluorescence intensity caused by the increase or decrease in the bacterial population. The *M*_i_ of the “hybrid” SH+Cu surface remains low even for densities up to 13.4 × 10^8^ colony-forming units per cm^2^ (CFU/cm^2^). Reproduced with permission from [187].

Ellinas et al. [187], observed that there is an upper bacterial concentration threshold in the antibacterial action of superhydrophobic surfaces and proposed a Cu enriched superhydrophobic surface as the “ultimate” “hybrid” solution to provide both short-term and long-term antibacterial efficacy. By making a direct comparison between the antibacterial properties and wettability of nanostructured superhydrophobic surfaces, flat metal-coated bactericidal surfaces of Ag and Cu, and “hybrid” nanostructured Cu bactericidal superhydrophobic (SH+Cu) surfaces, they found that Cu had a stronger antibacterial effect than Ag against a unicellular *Synechococcus* cyanobacterium, but both flat metal-coated surfaces lose their antibacterial properties for bacteria surface densities higher than 3.2 × 10^8^ per cm^2^, whereas both superhydrophobic (SH) and “hybrid” Cu-superhydrophobic (SH+Cu) surfaces are more antibacterial for surface densities up to 6.7 × 10^8^ per cm^2^, see Figure 15. Beyond this point, pristine SH surfaces show no antibacterial action and can only prevent bacteria adhesion, while the “hybrid” SH+Cu surfaces exhibit more potent antibacterial activity even for bacterial populations as high as 13.4 × 10^8^ per cm^2^. Overall, they demonstrate a scheme combining a reduced quantity of metal bactericidal agents with an anti-wetting surface that enables a highly advantageous antibacterial approach with reduced environmental risks.

There have been relatively fewer attempts to create superhydrophobic Ag-based antimicrobial surfaces. Cho et al. [190], studied the superhydrophobic and antimicrobial properties of Ag-plasma polymer fluorocarbon (Ag-PPFC) nanocomposite thin films fabricated using a ternary carbon nanotube-Ag-polytetrafluoroethylene (Ag-PTFE) composite sputtering target. The Ag nanoparticles of diameter 6–8 nm were found to be uniformly distributed in the PPFC matrix and the coating exhibited excellent water repellence due to the low surface energy of the PPFC matrix. The Ag-PPFC nanocomposite thin films were found to have superior antimicrobial properties, suppressing the growth and proliferation of *S. aureus* bacteria by up to 92.2% compared with uncoated substrates. This improved performance was attributed to the superhydrophobic property of the PPFC matrix combined with the antimicrobial characteristics of the Ag nanoparticles.

**Figure 16:**
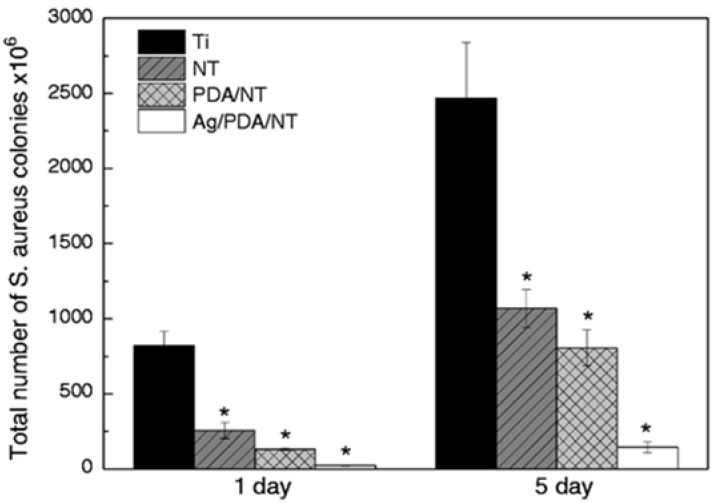
Total number of *S. aureus* colonies on different specimens after incubating for 1 and 5 days. (Data are shown as mean ± SD, *n* = 3, * represents *p* < 0.05 compared with Ti control surface). Reproduced with permission from [192].

Several other recent studies have focused on combining Ag with other contact-killing elements of Cu or TiO_2_ to create novel superhydrophobic antimicrobial surfaces. For example, Hong et al. [191], studied the combined antibacterial and superhydrophobic performance of Cu/Ag-doped multifunctional fabrics. They used Cu and Ag metal particles as antibacterial agents to create nanoscale roughness on the fabric surface and subsequently coated it with 1-dodecanethiol to produce a superhydrophobic surface. The Cu/Ag treated and Cu treated fabrics showed higher antibacterial rates ≥ 99% against Gram-positive *S. aureus* than the Ag treated fabric. Moreover, fabrics treated with Cu and Cu/Ag particles and with a hydrophobic coating displayed superhydrophobic characteristics with contact angles of 161–162°. Zhang et al. [192], presented a novel strategy to create a hierarchical antibacterial coating by combining the bactericidal nature of nano-patterned topography TiO_2_ nanotubes (NT), polydopamine (PDA), and Ag nanoparticles. Anodized TiO_2_ NTs and self-polymerized PDA were both used as preliminary antibacterial agents and gave a significant reduction in the number of *S. aureus* colonies after 1 and 5 days of incubation. At the same time, the storage capacity of the nanotubes and the in-situ reduction activity of polydopamine were used to introduce large amounts of strongly attached Ag nanoparticles to further enhance the antibacterial performance of the Ag/PDA/NT coatings as compared to the pristine Ti control surface, see Figure 16. 

**Figure 17:**
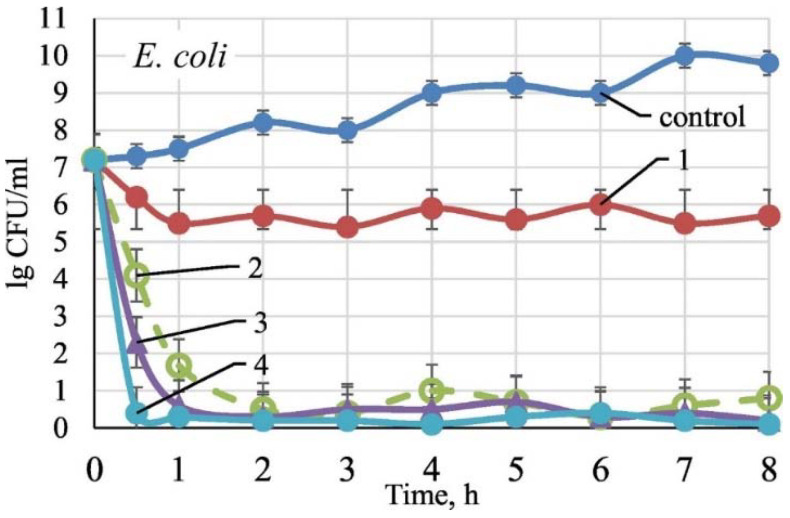
*E. coli* growth inhibition in presence of: 1—TiO_2_/SiO_2_; 2—TiO_2_/SiO_2_/Ag_9.7_ at.%; 3—TiO_2_/SiO_2_/Ag_14.4_ at.%; 4—TiO_2_/SiO_2_/Ag_19.8_ at.%; control—in absence of sample. Reproduced with permission from [194].

Gorguluer et al. [193], deposited superhydrophobic, antimicrobial, self-cleaning Ag nanoparticle TiO_2_-PDMS coatings on fabrics using a simple dip-coating method. Their results showed that the superhydrophobic performance of the coating was improved by combining the rough surface structure of the Ag and TiO_2_ nanoparticles with the low surface energy of PDMS. The Ag nanoparticles also demonstrated strong antibacterial activity against *E. coli* and *S. aureus*. Vladkova et al. [194], used magnetron co-sputtering to deposit triple TiO_2_/SiO_2_/Ag nanocomposite thin films with expected antimicrobial activity. The films demonstrated a strong inhibitory effect toward *E. coli* growth with the number of viable bacterial cells approaching zero in the first 30 min to 1 h. However, this strong antimicrobial activity was shown to depend insignificantly on the surface wettability, surface energy, and topography on the base TiO_2_/SiO_2_ structure and instead, is determined by the presence of the antimicrobial agent Ag and its concentration in the coating, see Figure 17.

There has also been an attempt to create a ZnO-based superhydrophobic surface by Valenzuela et al. [195], who studied the dual-action self-cleaning and antimicrobial properties of photoactive electrosprayed ZnO nanoparticle coatings. The surfaces were found to have an excellent photoactive performance which kept the coatings free from bacterial colonization and biofilm formation and strong antibacterial activity against *S. aureus*, with a >99.5% reduction in the number of culturable cells. The biocidal activity was attributed to the photogenerated ROS on the surface of ZnO coatings and the bioavailable Zn ions produced from ZnO dissolution.

**Figure 18:**
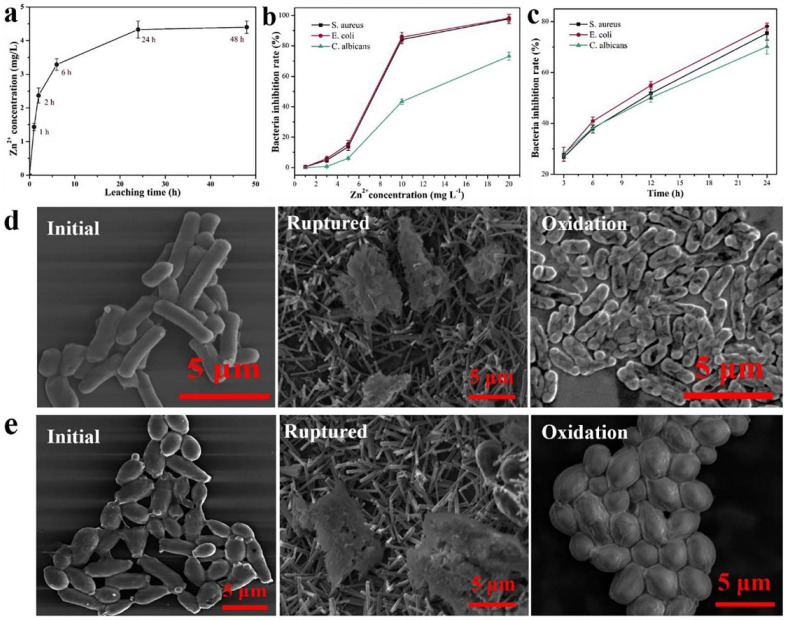

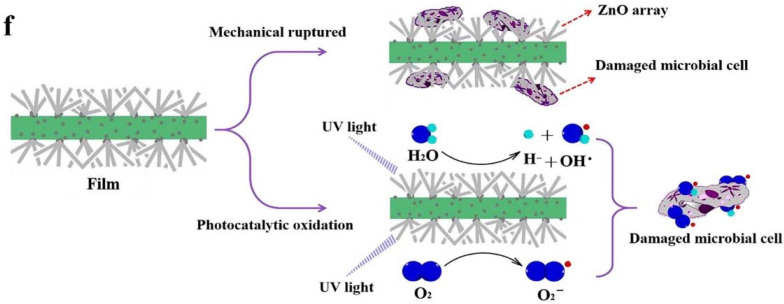
(**a**) Zn leached from ZnO nanoparticle Zn^2+^/Cel-6 film at different times. (**b**) bacteria inhibition rate of different Zn^2+^ concentrations for *S. aureus*, *E. coli,* and *C. albicans*. (**c**) bacteria inhibition rate of ZnO nanoparticle Zn^2+^/Cel-6 film not treated by UV for *S. aureus*, *E. coli,* and *C. albicans*. SEM images of (**d**) *E. coli* (bacteria) and (**e**) *C. albicans* (fungus) cells before and after mechanical rupture (without UV treatment) and photocatalytic oxidation inactivation of ZnO nanoparticle Zn^2+^/Cel-6 film respectively. (**f**) Schematic of antimicrobial mechanism of ZnO nanoparticle Zn^2+^/Cel films. Reproduced with permission from [196].

There have been relatively fewer investigations into the combined antimicrobial effects of chemical contact-killing surfaces and physical surface nanoprotrusions and these studies have been focused on ZnO-based materials [196,197]. A very recent study by Xie et al. [196], investigated the antimicrobial properties of Zn cellulose-based films incorporating ZnO nanopillars. The in-situ formed ZnO crystal nucleus on the cellulose-based film provided a firm site for the growth of ZnO nanoparticles, which offered significant antimicrobial activity against both Gram-positive and Gram-negative bacteria as well as fungus. The antimicrobial mechanism was shown to depend on UV excitation. Films without UV excitation inactivated the microbial cells mainly through mechanical rupture induced by ZnO nanoparticles; whereas for films with UV excitation, inactivation was via the synergistic action of photocatalysis and mechanical rupture, thus leading to Zn/Cel (cellulose) coatings with high antimicrobial efficiency and long-term effectiveness, see Figure 18. Carvalho et al. [197], investigated the combined influence of film thickness, surface morphology, and Ag doping on the antimicrobial performance of ZnO coatings prepared by DC reactive magnetron sputtering. They found that the Ag-doped ZnO coatings had V-shaped columnar structures which increased in surface area with an increase in coating thickness. Samples with film thicknesses in the range 200–600 nm gave better antibacterial activity against *E. coli* than thinner samples of 50 to 100 nm and this was further improved with increasing Ag content in the ZnO coatings. 

**Figure 19:**
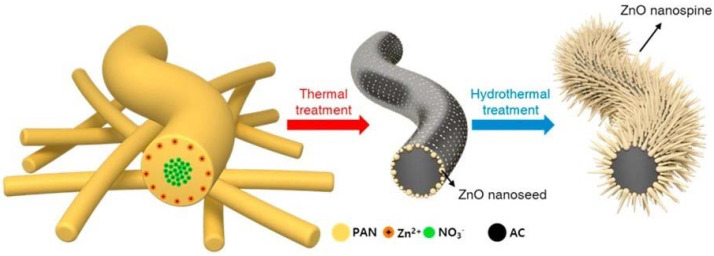
Schematic diagram illustrating the synthesis procedure of ZnO nano-spines on an activated-carbon nanofiber (ZnO/ACNF) structure. Reproduced with permission from [198].

To date, there have been no previous attempts to combine all three microorganism-killing phenomena of chemical contact-killing surfaces, superhydrophobic surfaces, and physical surface nanoprotrusions in a thin film coating format. However, a very recent study by Kang et al. [198], attempted to grow hierarchical ZnO nano-spines on an activated-carbon nanofiber (ACNF) layer for efficient airborne virus and bacteria inactivation. They used thermal/hydrothermal treatment to produce a highly-distributed nanoseed layer of sharp ZnO nanospines with enhanced hydrophobicity and excellent antimicrobial performance by the synergistic effect of physical and photocatalytic oxidative stress toward a wide range of bacteria and viruses, see Figure 19.

### Anti-SARS-CoV-2 Coatings

Another important area that is receiving increasing attention is the advancement of antiviral coatings to fight the deadly COVID-19 disease [199]. Researchers have been working throughout the current pandemic to develop the next generation of antimicrobial materials to reduce the spread of infection via high touch surfaces as well as PPE surfaces [4], which account for around 20% of total infections among healthcare workers [200].

Anti-SARS-CoV-2 coatings of Cu Ag and TiO_2_ are being continuously researched and developed. Hutasoit et al. [201] assessed the antiviral performance of cold sprayed Cu particle (5 to 60 µm) coatings on stainless steel, with and without annealing. They found that both as-deposited and annealed samples were able to reduce the infectivity of the SARS-CoV-2 virus by 99.2% and 97.9%, respectively, when it was left on the surface for 5 h. Behzadinasab et al. [202] evaluated the anti-SARS-CoV-2 properties of Cu_2_O coatings on glass and stainless steel. The coating was found to inactivate >99.9% of the virus within 1 h and remained as potent after 5 exposure cycles and the following storage in water for 2 weeks and disinfection with 70% ethanol. Hosseini et al. [203] fabricated a hydrophilic, porous CuO coating with a large surface area to draw in and rapidly deactivate SARS-CoV-2. Their results showed that a 30 μm thick Cu_2_O coating is able to reduce the virus infectivity by 99.8% in 30 min and below the detection limit after 1 h. Balagna et al. [204] undertook preliminary studies into the virucidal effect of Ag nanocluster/silica composite sputtered coatings against the SARS-CoV-2 virus on the surface of facial masks. Their study showed that the coating can be deposited on practically every kind of filtering media and is able to completely reduce the titre of SARS-CoV-2 to zero. Kumar et al. [205] also investigated the potential of photodeposited Ag nanoparticles to destroy SARS-V-2 on the surface of fabrics. The fabrics were shown to be durable during washing cycles and exhibit strong antimicrobial performance, with 97% annihilation of the SARS-CoV-2 virus. Khaiboullina et al. [91] demonstrated that UV light exposure of photoresponsive TiO_2_ nanoparticle-based coatings could be potentially used to destroy Covid-19 viral particles. Their preliminary results showed that the genomic RNA of HCov-NL63 cells (a close genetic relative of SARS-CoV-2) were completely degraded after 30 min of UV exposure. Micochova et al. [206] also evaluated the efficacy of light-activated TiO_2_ and TiO_2_–Ag coatings prepared by spray gun on ceramic tiles, on the infectivity of SARS-CoV-2. They showed that the percentage of the infected SARS-CoV-2 after 1 h was only 15% on the illuminated TiO_2_ coating compared to 80% on a polystyrene surface. They also reported that introducing Ag into the TiO_2_ coatings did not give any further improvements in their antiviral activity.

Recently, Anti-SARS-CoV-2 materials containing antimicrobial metals have also been produced by additive manufacturing (AM) [207], which is another promising advanced technique to develop multi-functional layer-by-layer coatings to prevent implant-associated infections [208,209,210]. Robinson et al. [207] proposed the use of AM combined with surrogate modelling for the rapid manufacture of a novel Cu-W-Ag microporous architecture to fight SARS-CoV-2. They found that microporous Cu-W-Ag with average pore sizes of 80 µm gave 100% inactivation of a biosafe enveloped ribonucleic model of SARS-CoV-2 within 5 h. Work by the same group [209] investigated the novel use of the laser powder bed fusion AM technique to print a porous Co-Cr-Mo superalloy with potent antiviral activity, demonstrating a reduction in inactivation time of SARS-CoV-2 from 5 h to 30 min. Amin Yavari et al. [210] developed multi-functional layer-by-layer chitosan-based coatings on the surface of porous Ti to simultaneously prevent implant-associated infections and stimulate bone tissue regeneration. The developed meta-biomaterials showed strong antibacterial behaviour with no signs of biofilm formation and up to 8 orders of magnitude reduction in bacteria. 

## 6. Summary

There is increasing global interest in antimicrobial high-touch surfaces with the potential to significantly reduce the lifetime of deadly microorganisms such as SARS-CoV-2. This article has reviewed recent advances in metal-based coatings for such antimicrobial high-touch surfaces with a specific focus on three discrete microorganism-killing phenomena of contact-killing surfaces, nanoprotrusions, and superhydrophobic surfaces. 

The antimicrobial effects of chemical contact-killing surfaces made from metals such as Cu, Ag, Zn, and TiO_2_ were reviewed in detail, and examples of where these elements have been combined to create binary and ternary coatings were discussed. Environmentally benign TiO_2_ photocatalytic coatings with active Cu and Ag ingredients were highlighted as one of the best options to provide long-term antimicrobial efficiency with anti-corrosion surface features. Next, the self-cleaning and bacterial resistance of purely structural superhydrophobic surfaces were reviewed. It was found that superhydrophobic nanoporous and nanopillared features could be effectively engineered on the coating surface to give significant reductions in bacteria attachment. The potential of physical surface nanoprotrusions to damage microbial cells on the coating surface was also considered. Recent studies based on TiO_2_ surface nanopillars have revealed their potential multifactorial killing approach is based on a combination of nanopillar-induced cell impedance and oxidative stress which impair bacterial growth and biofilm formation leading to time-dependent reductions in bacterial viability.

Finally, recent attempts to combine these individual microorganism-killing phenomena were considered. It was found that the antimicrobial performance of TiO_2_ superhydrophobic surfaces was significantly improved by introducing a biocidal contact-killing element like Cu, Ag, or Zn. This solution has been proposed to give both short-term and long-term bacteria-killing efficiency with the potential to reduce the level of potentially toxic biocidal chemical agents. There have been relatively fewer studies on the effects of combining physical surface nanoprotrusions with chemical contact-killing surfaces or superhydrophobic surfaces. However, one very recent study has highlighted the potential of ZnO surface nanospines with enhanced hydrophobicity and excellent antimicrobial performance due to the synergistic effect of physical and photocatalytic oxidative stress. 

Looking ahead, although chemical contact-killing surfaces of Cu, Ag, and Zn have proven antimicrobial performance, they can lead to bacterial resistance and become ineffective over time. Superhydrophobic coatings offer a purely structural solution that does not induce bacterial resistance but there is a limit to their antimicrobial action. Surface nanoprotrusions have also shown potential to effectively kill bacteria but their long-term antimicrobial performance remains largely unexplored. There is clearly potential in combining these three microorganism-killing phenomena but more focused research is required to truly understand their synergistic effects. It is expected that the next generation of super-antimicrobial metal-based coatings will combine a nanosurface texture that can effectively repel the majority of microorganisms with surface nanoprotrusions that can kill any that remain attached. This could be further enhanced with chemical contact-killing elements to provide a triple layer of antimicrobial protection.

## Figures and Tables

**Table 1:** Common synthesis methods to produce Cu, Ag, ZnO, and TiO_2_ nanoparticles and their corresponding advantages and disadvantages. Data adopted from [105,106,107,108].

Element	Synthesis Type	Methods	Advantage	Disadvantage	Further Reading
Cu	Chemical methods	Chemical reaction/reduction	Simple, Versatile, Saleable	Toxic and Flammable chemicals	[109]
		Microemulsion/colloidal method	Simple, high control, homogenous size	Expensive, surfactants lower yields	[110]
		Sonochemical method	Low cost, safe, environment friendly	Agglomerations, lower yield	[111]
		Electrochemical method	Low cost, accessible instrumentation	High current densities	[112]
		Hydrothermal decomposition	High yields, small size, high purity	Complex control on process	[113]
	Physical methods	Pulse laser ablation/deposition	Clean process and scalable	Expensive and uniformity challenges	[114]
		Pulsed wire discharge method	Fast process, high purity	Limited production, contaminations	[115]
		Mechanical/ball milling method	Efficient and low cost	Long process	[116]
	Biological synthesis	Green methods	Green synthesis, localized nanoparticles formation	Little knowledge available	[117]
Ag	Chemical methods	Sol-gel process	High purity, uniform size	Expensive precursors, scalability	[118]
		Reverse micelle	Simple and scalable	Lower yields, Toxic and hazardous chemicals	[119]
		Chemical vapor deposition (CVD)	One-step process	High cost, complex process, scalability issues	[120]
		Wet chemical synthesis	Simple process and small particle size	Toxic and hazardous chemicals	[121]
	Physical methods	Arc discharge	Simple, process, high yield, high purity	Large size distributions	[122]
		Laser ablation	Small size particles, high purity	Higher energy consumptions, lower yields	[123]
		Evaporation/condensation	Industrial-scale synthesis	Expensive instrumentation and process	[124]
		Ball milling	Simple process	Small-scale process, agglomerations	[125]
	Biological synthesis	Bacteriogenic synthesis	Simple and eco-friendly	Slow production, pathogenic behaviours	[126]
		Fungi based synthesis	Simple, fast, and high intakes	Long process, pathogenic behaviours	[127]
		Virus driven synthesis	Simple, environmentally friendly, small size	Less metal-binding sites, expensive requisites	[128]
		Algae driven synthesis	Simple and low-cost	Slow production	[129]
		Plant-based synthesis	Non-pathogenic and non-hazardous compounds	Little knowledge of unknown mechanisms	[130]
ZnO	Chemical synthesis	Hydrothermal synthesis	High purity	Lower yields	[131]
		Sol-gel method	Control over morphology	Lower yields	[132]
		Direct precipitation method	Uniform size	Purity issues	[133]
		Sonochemical method	Facile and environmentally friendly	Difficult scalability	[134]
		Solvothermal method	No calcination prerequisites	Time-consuming process	[135]
	Thermal process	Thermal catalyzation	High production efficiency	Health and safety protocols	[136]
		Combustion method	High purity	High temperature	[137]
	Physical methods	Mechanochemical process	High purity	High cost	[138]
		Microwave irradiation methods	Simple and economical process	Difficult scalability	[139]
		Laser ablation method	High energy inputs	Lower yields	[140]
	Biological synthesis	Plant-based reduction agents	Above 95% conversion to nanoparticles	Little knowledge of unknown mechanisms	[141]
TiO_2_	Chemical synthesis	Sol-gel method	High conversion rates	Difficult to scale-up	[142]
		Ionic-liquid assisted synthesis	Simple process, atmospheric conditions	Expensive raw materials	[143]
		Microemulsion technique	thermodynamic stable, controlled size growth	agglomeration	[144]
		Precipitation method	Controlled process	Temperature- dependent crystallinity	[145]
		Electrochemical synthesis	Versatile and low-temperature	Required high energy inputs	[146]
	Thermal synthesis	Hydrothermal method	Homogeneous particles size	Usage of multiple chemicals	[147]
		Solvothermal method	Usage of organic solvents	Crystal size is sensitive to precursor composition	[148]
	Biological synthesis	Green synthesis	eco-friendly, mild conditions	toxic chemicals	[149]
